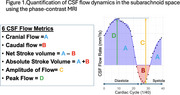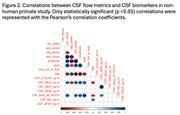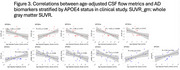# APOE ε4 Modulates Beta‐Amyloid Clearance via Cerebrospinal Fluid Dynamics: Insights from Nonhuman Primate and Clinical Studies

**DOI:** 10.1002/alz70856_104986

**Published:** 2026-01-08

**Authors:** Jeongchul Kim, Megan E. Lipford, Richard A. Barcus, Hongyu Yuan, Qing Lyu, Jeremy Patton Hudson, Sam N. Lockhart, Timothy M. Hughes, Courtney L. Sutphen, Brett M. Frye, Kiran K. Solingapuram Sai, Marc D. Rudolph, Carol A. Shively, Thomas C. Register, Michelle M Mielke, Suzanne Craft, Christopher T Whitlow

**Affiliations:** ^1^ Wake Forest University Health Sciences, Winston Salem, NC, USA; ^2^ Wake Forest University School of Medicine, Winston‐Salem, NC, USA; ^3^ Wake Forest School of Medicine, Winston‐Salem, NC, USA; ^4^ Atrium Health Wake Forest Baptist, Winston‐Salem, NC, USA; ^5^ Wake Forest School of Medicine, Winston Salem, NC, USA; ^6^ Atrium Wake Forest Baptist, Winston‐Salem, NC, USA; ^7^ Wake Forest Alzheimer's Disease Research Center, Winston‐Salem, NC, USA; ^8^ Emory and Henry University, Emory, VA, USA; ^9^ Division of Public Health Sciences, Wake Forest University, School of Medicine, Winston‐Salem, NC, USA

## Abstract

**Background:**

Efficient cerebrospinal fluid (CSF) circulation plays a critical role in clearing metabolic waste, including beta‐amyloid (Aβ), a key biomarker of AD. Impaired CSF dynamics may contribute to amyloid accumulation and disease progression. To elucidate clearance mechanisms, we investigated the relationship between subarachnoid CSF flow dynamics—measured via phase‐contrast magnetic resonance imaging (PC‐MRI)—and amyloid burden in both a nonhuman primate (NHP) aging cohort and the Wake Forest ADRC clinical cohort.

**Methods:**

CSF flow dynamics were quantified across the cardiac cycle in the pontine cistern, cerebellomedullary cistern, cerebral aqueduct, and spinal canal (Figure 1). The NHP study included 16 female vervet monkeys (ages 10–27 years), assessing CSF flow in relation to age and CSF biomarkers. The clinical study analyzed 47 participants (31 cognitively normal, 14 with mild cognitive impairment, and 2 with dementia) recruited from the ADRC Clinical Core. CSF flow metrics were correlated with amyloid positron emission tomography (PET) imaging and apolipoprotein E (APOE) genotype to evaluate clearance differences between APOE ε4 carriers and non‐carriers.

**Results:**

In the NHP cohort, CSF flow metrics showed strong negative correlations with age (Spearman's ρ = ‐0.68 to ‐0.83) and positive correlations with the CSF Aβ42/40 ratio (ρ = 0.67 to 0.84) (Figure 2), while plasma biomarkers exhibited no significant association with CSF flow. Age‐adjusted analyses confirmed moderate correlations between CSF flow and CSF Aβ42/40 (ρ = 0.45 to 0.75). In the clinical cohort, APOE4 carriers exhibited reduced CSF flow dynamics with greater amyloid burden, suggesting impaired clearance. Conversely, non‐carriers demonstrated increased CSF flow dynamics in response to amyloid accumulation, indicating distinct compensatory clearance mechanisms (Figure 3). Regional associations were observed between CSF flow in the pontine cistern and anterior gray matter standardized uptake value ratio (SUVR) on amyloid PET, as well as between CSF flow in the cerebellomedullary cistern and posterior gray matter SUVR.

**Conclusion:**

These findings underscore heterogeneity in amyloid clearance mechanisms between APOE4 carriers and non‐carriers, highlighting the importance of integrating CSF flow dynamics into stratified diagnostic and therapeutic strategies. While NHP models provide valuable insights into age‐related CSF flow decline, species‐specific differences in APOE4 non‐carriers emphasize the need for clinical validation in human populations.